# Indonesia's Unique Social System as Key to Successful Implementation of Community- and Home-Based Palliative Care

**DOI:** 10.1200/GO.22.00290

**Published:** 2023-06-08

**Authors:** Venita Eng, Aru W. Sudoyo, Siti A. Nuhonni, Kevin Hendrianto

**Affiliations:** ^1^ Indonesian Cancer Foundation Jakarta Chapter, Jakarta, Indonesia; ^2^ Indonesian Cancer Foundation, Jakarta, Indonesia; ^3^ Medical Faculty, Universitas Pelita Harapan, Tangerang, Indonesia

## INTRODUCTION

Globally, the cancer burden is increasing with significant morbidity and mortality. In 2020, there were more than 19.3 million new cancer cases and 10 million cancer-related deaths worldwide. Studies have shown that the number will increase at an alarming rate, reaching an estimation of 29.4 million cases in 2040.^1,2^ There continues to be significant disparities between higher- and lower-income countries, where the burden is greatest in the latter, with a rapid increase in cancer incidence, morbidity, and mortality.^1-4^

**CONTEXT**

**Key Objective**
Community- and home-based palliative care is essential to ensure access of care for patients with cancer. This study aims to give insight from Indonesia's practice and strategies in palliative care development.
**Knowledge Generated**
In large diverse population, key for implementation is multilayer strategic collaboration from policy stakeholders, professionals, nonprofit organizations, and empowering volunteers from community.
**Relevance**
Points of strategies from Indonesia may be applied in other countries with similar traits of development in large diverse community.

The number of cancer cases found in the advanced stage, along with their burden and suffering, signifies the need for good palliative care. One of the Union for International Cancer Control (UICC) four key pillars of advocacy to address global equity gap in cancer care is ensuring at a minimum a basic supportive and palliative care service for all.^2^ ASCO also has published multiple statements to support the integration of palliative care to enhance benefits to patients across cancer journey.^5^ However, from 40 million people globally in need of palliative care, just 14% receive it, predominantly in high-income countries.^6^

Indonesia is the largest archipelago country in the world in its development stage and has an urgent increasing burden of cancer (1.8 every 1,000 people), with the majority of cases (70% from 240,000 incidence per year) found in the advanced stage.^7-9^

Considering the cancer burden in the advanced stage, it was estimated in 2018 that 662,261 Indonesian people are in need of palliative care support.^7,8,10^ Accordingly, where the need for palliative care is not met, patients with terminal and incurable illnesses suffer total pain and burdensome costs.^6^ Also, in the terminal stage, patients would opt for home care for end-of-life management in consideration of cost and family ties and thus the need for good home-based palliative care in the community.^11^

As a challenge, Indonesia has a large population (240 millions people, fourth largest in the world) and a geographical status of 13,466 island and 37 provinces which spread between 1.3 million km.^12^ This large area and variance in geographical and topographical characteristics between provinces result in cultural and custom differences between provinces.^13^

In terms of strength, Indonesia has advantages in the community to aid in cancer and palliative care management, including the Indonesian Cancer Foundation (ICF), a uniquely tiered population system, active community participation through health care volunteers, and the integration of resources in the national health care system.

## INDONESIAN CANCER FOUNDATION

ICF (*Yayasan Kanker Indonesia*) is a nonprofit organization with social and humanity vision in health care, especially in cancer care. The ICF's mission was to increase community awareness and participation in cancer care. The ICF programs aim to support cancer care through promotive, preventive, supportive, and palliative care. Currently, the ICF has 114 branches throughout Indonesia and serves as the largest cancer foundation network.^14^

Considering that cancer care would only thrive with multisectoral involvement, the ICF would conduct its services through cooperation with multiple stakeholders: professional organizations, NGO, and the private and business sectors, nationally and internationally. Additionally, unique to other foundations, the ICF has strong ties with the government, including the governor's leadership role. Thus, the program is in conjunction with assisting local governments with cancer care. The ICF plays a role in filling the gap between government health care programs and community needs.^14^

Since 1995, the ICF has received international palliative care training and started home hospice care programs to increase cancer patients' quality of life and provide dignity for quality of death. In accordance with the WHO guidelines for the implementation of home-based palliative care, the ICF has set up a team of doctors, nurses, and volunteers to serve vulnerable patients at home.^15,16^ For 12 years since its conception, the ICF has served 621 patients in which 79% of them passed peacefully at home after a median of 1-2 months of care with the ICF palliative team.^15^ The service continues until now (2022), with an average of 80 services per year, including the development of hospice buildings in South Jakarta.^14^

In addition to direct service to patients, the ICF also implements the WHO guidelines for community-based palliative care by providing public education and socialization of programs in conferences.^15,16^ From 2015 to 2022, the ICF has conducted palliative care training for caregivers in the community, volunteers, and health care professionals in 10 provinces, with a total of 2,353 participants receiving training. Some of these trainings are joint collaborations with the local district of health and international foundations.^17^

In 2015, an attempt to integrate home palliative care with the government and primary health care system was also made by the ICF Jakarta chapter.^18^ This is in consideration of Daerah Khusus Ibukota (DKI) Jakarta as a capital city with a significant and increasing burden on patients with cancer, including nationwide patients coming to Jakarta for advance treatment.^19^ Furthermore, there was congestion at the hospital level, partly because of the unavailability of adequate home care/community-based palliative services.^18^

The ICF Jakarta Palliative call center and palliative home care service were initiated to allow patients with terminal disease to be discharged early and prevent hospital readmission with good-quality home care services. Palliative call center service includes 24-hour patient consultation with trained nurses and doctors. After referral from the hospitals, patient care was continued at home until death and bereavement.^18^

Bereavement services could start in hospitals from anticipatory grief moments, identification of potential complicated grief, and follow-up at home by the ICF team, either by phone calls or direct visitations. The providers of bereavement services include hospital counseling/chaplaincy teams and volunteers trained by the ICF.^18^

In the 2022 report, the ICF Jakarta palliative service has served 355 patients, with the majority (67%) passing away at home, mitigating the tendency for patients to stay in the hospital until dying.^18^ This finding aids in reducing hospital congestion and improving cancer care quality in Jakarta, Indonesia. A multidisciplinary team from the hospital and community is arranged, from specialists to social workers and volunteers, to ensure maximum comfort for the patient.^19^ During COVID-19 pandemic in 2019-2022, ICF Jakarta continued its service and served 731 teleconsultation services and remote delivery of symptomatic medicines to ensure that patients at home still received essential palliative care service.^20^

ICF Jakarta collaborates with the District of Health government in care provision and education, ensuring regional coverage and setting a principal model for other regions in Indonesia. Concurrently, ICF Jakarta also trained community health care volunteers (CHVs) in cancer prevention and early detection as well as in palliative care provision in the community. In 2015-2022, ICF trained 611 volunteers in palliative care in collaboration with the community family welfare program.^20^ In the consequent years, 100 of these volunteers received further advanced training and refreshment courses for practical issues in home and community-based palliative care. These community volunteers serve as extensions of direct care in the neighborhood.^20^

Standard operating procedures for health care professionals and volunteer joint work differ according to each health care center policy. In Jakarta, volunteers may report findings of patients in need of medical attention to local health care centers and vice versa; the health care center may ask for volunteers' help in follow-up for the patient's condition. The ICF has regular volunteer meetings for training and refreshment, which also serve as an opportunity for consultation with health care professionals. At times, joint home visits are conducted, for example, with a patient whose volunteers know earlier for better acceptance of medical professionals visiting home. Additionally, health care professionals may perform joint visits to introduce volunteers to patients to assist in future companionship and/or follow-up visits.^20^

## INDONESIA UNIQUE POPULATION SYSTEM AND ITS ADVANTAGE

**FIG 1:**
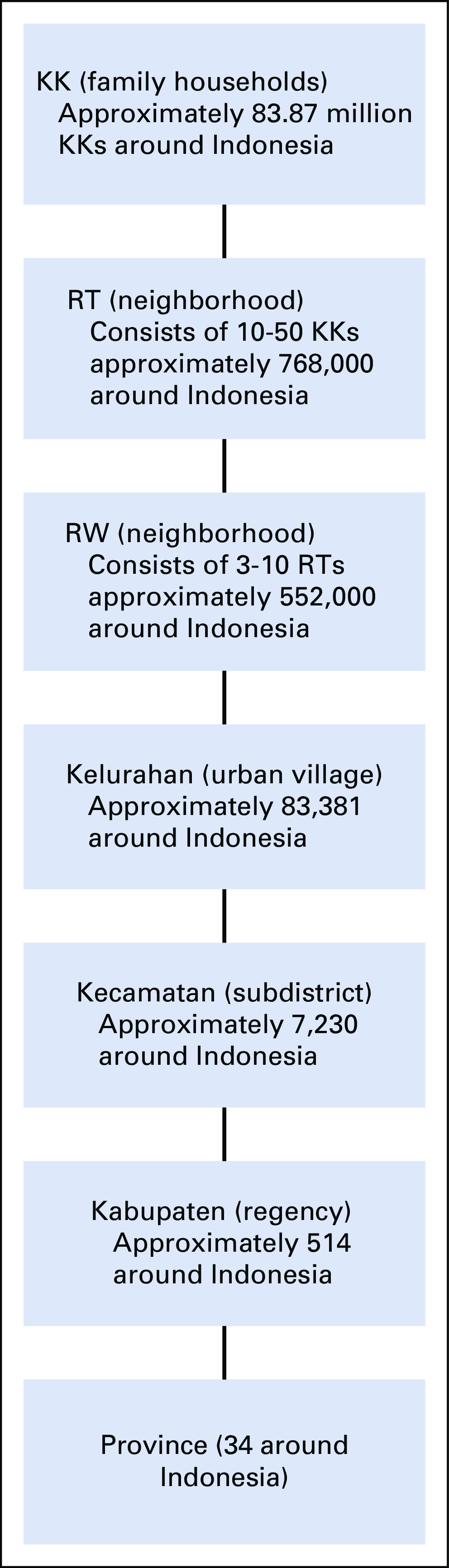
Population system in Indonesia. KK, Kartu Keluarga; RT, Rukun Tetangga; RW, Rukun Warga.

Indonesia has a unique population system that allows for effective implementation of health care and social support for the smallest family unit. Indonesia's population system is tiered from the smallest unit of family to Rukun Tetangga (RT/neighborhood), Rukun Warga (RW/large neighborhood), Kelurahan (urban village), Kecamatan (subdistrict), Kabupaten (regency/municipality), and province. According to the Department of Population and Civil Registry Office, Ministry of Home Office data, and Indonesia's administrative areas, there are 768,000 RTs (neighborhood), 552,000 RWs (neighborhood), 83,381 Kelurahan (urban village), 514 Kabupaten (regency), and 37 provinces (Fig 1).^21^

The tiered system allows each household and family to be monitored, improves service delivery, protects community safety and stability, connects people to the higher government, allows education and information dissemination, improves quality of life, allows community initiative and active participation, and optimizes each person's role in community welfare through mutual assistance.^22^

Indonesia has an ideology of collective, cooperative, and consensual social interaction, called the philosophy of *Gotong royong.* In literal terms, *gotong royong* is mutual assistance, which signifies social solidarity within the smaller bubbles of the community. *Gotong Royong* is seen as an obligation of an individual toward society with the aim of sharing burdens among the members of the community, thus building trust, friendships, and preventing conflicts.^23^

*Gotong royong* is practiced in many social activities, such as traditional communal/cooperative labor work to clean the neighborhood environment or gathering events such as weddings by donating funds, food, or labor. Consequently, in the future, the receiving neighbor will repay the next member of the community in need by holding similar responsibilities and tasks. This interaction also includes and is not limited to infrastructure building, irrigation, and clearing fields for crops. The government and military forces have also been known to use this communal labor for disaster relief.^23,24^

In the context of cancer and palliative care, the tiered neighborhood system and *gotong royong* mutual assistance serve as valuable tools for early detection and ensuring continuous care throughout the cancer journey. For example, neighbors would help recognize patients or families in need of cancer care and referral to a higher level. When a patient returns home after treatment or during the terminal stage, community members can help with basic care and daily needs of patients and their families, as well as companion and social support.

In the spirit of outreach health care to households in need, the district of Health in Jakarta also implements the family approach through a program called *Ketuk Pintu Layani Dengan Hati* (KPLDH) or literally translated as knocking door and serving with heart. Since 2015, 481 teams have been established, consisting of a doctor, nurse, and midwife, who are responsible for 5,000 person in the community.^25,26^

KPLDH was endorsed by government decree to aim at improving health services for the community by supporting and assisting activities of the primary health care center (*Puskesmas*). The KPLDH program involves carrying out comprehensive promotive, preventive, and supportive efforts to overcome health problems and achieve optimal public health.^26^

## INDONESIAN CHVs

The national guidelines for palliative cancer care describe volunteers as an integral part of the palliative care team whose role may vary on the basis of needs. CHVs in Indonesia or *Kader* are trusted individuals from local communities who help health care professionals increase patient and family quality of life through education, direct care, increasing awareness, fundraising, and assisting in rehabilitation.^27-29^ Volunteers play a role in improving community access to palliative care and filling in the gap of health care provider scarcity across service levels.^27^

**FIG 2:**
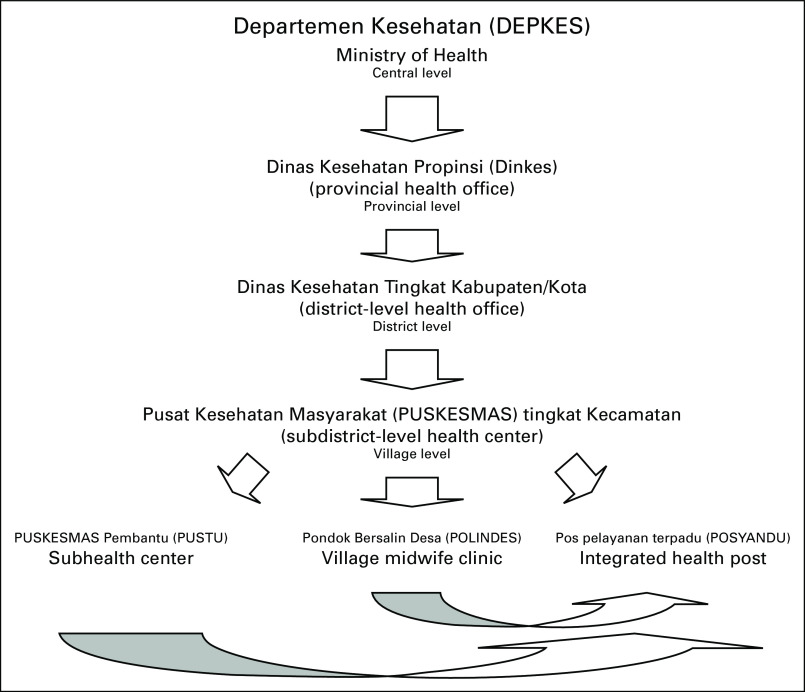
Tiered health care system in Indonesia.^27^

*Kader* system started in 1970 with the National Women's Family Welfare Movement (*Pemberdayaan Kesejahteraan Keluarga*) endorsed by the Ministry of Home Affairs. They were initially trained to conduct health and nutrition promotion activities in each village. *Kaders* are chosen by and from within the community to support a health post in a community called *posyandu* with supervision from medical professionals.^30^ The relationship between multiple-level health posts is shown in Figure 2.

*Posyandu* and its *kaders* provide a foundation for health care outreach in modern Indonesia. Despite the vulnerabilities of modernization, the *posyandu* system in Indonesia, run by volunteers for 5 decades, is probably the largest and longest continuous community-based volunteer health program in the world with 1.5 million *kaders* covering 86% of Indonesia.^27^ *Kaders* are manifestations of community participation in joint efforts to solve health problems in their areas, thus playing a respectable and vital role in fair and equitable national health.^31^

Studies have shown promising results in evaluating *kader* effectiveness of *kaders* in cancer and palliative care programmes. A study by Saragih in 2020 found that education and involvement of community volunteers in palliative care provision in Medan had a positive impact on patients' quality of life.^32^ Another report in Bangli, a village in Bali, showed community initiative to form a program to initiate education and the establishment of village health care volunteers for reproductive health, including cervical cancer prevention, as it is related to poor socioeconomic status and a high number of underage marriages. The program was coordinated by village midwives, *puskesmas*, and local non-government organization (NGO).^33^

During the COVID-19 pandemic, when people postponed regular cancer screening programs, the community in Semarang, Central Java, initiated training for *kader* to enhance awareness of cervical cancer as it has been shown that education is related to attitudes toward cancer screening. This shows the valuable role of community participation in mitigating the pandemic's effect on cancer control.^34^

In palliative care, community Gresik, East Java, provides training and assistance to develop women's capacity for palliative care services in villages. Women were chosen for the program for several reasons, including empathy, better social relationships with people in the community, sensitivity to emotional issues, and listening capacity. Palliative care services conducted by volunteers focused mainly on psychosocial and spiritual support to motivate patients, increase understanding of the disease, and empower patients and families.^35^

Volunteers are not trained or certified medically, limiting their focus on social support. However, they received informal training regarding their patients' needs and how to provide psychosocial support. One of the providers of the training is the ICF, which has a regular training program for caregivers and public laypersons. The training is conducted by health care professionals, and in their role, volunteers are overseen by primary health care centers according to their area of service.^20^

## INTEGRATION OF RESOURCES IN NATIONAL HEALTH CARE SYSTEM

Community resources and initiatives have been integrated into government policy. In 2007, the Ministry of Health addressed palliative cancer programs as an urgent humanity need in Indonesia and must be integrated into the health care system at all levels, especially community- and home-based care with involvement of the public and private sectors, adapting to local specific cultural, social, and economic conditions.^11^ This is also in accordance with the WHO recommendation for the development of palliative care as an integral part of continuum cancer care.^36^

**FIG 3:**
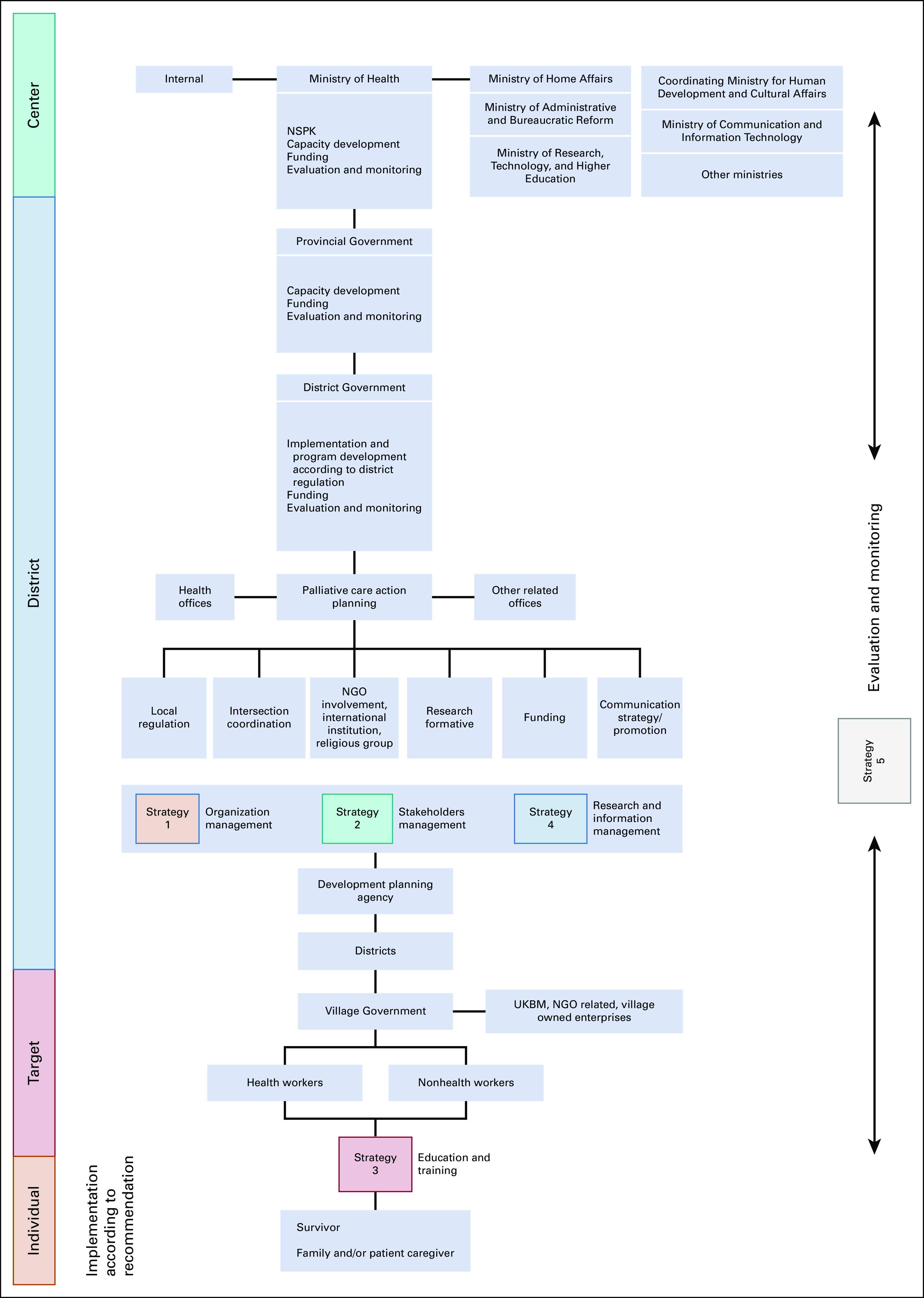
Flow diagram of palliative care implementation plan.^8^ NGO, non-government organization; NSPK, Norma, Standar, Prosedur, Kriteria; UKBM, Upaya Kesehatan Berbasis Masyarakat.

National guidelines emphasize the need to ensure a systematic, tiered referral system. The referral system ensures the timely management of patients and cost control. Unique to cancer palliative care program, back referral from hospital to community/primary health care level is mandatory to ensure patients receive good quality and continuation of palliative care at home.^11^ National Cancer Control Committee/*Komite Penanggulangan Kanker Nasional* describes the relationship, roles, and referral system from the center to the individual in Figure 3.^8^

The National Care Management Program includes guidelines for human resource education and allocation at each level, quality standardization, promoting funding, facilitating volunteer and community participation, information dissemination, and increasing overall access in the community for quality palliative care.^11^ Each level of health care centers differs in human resource allocation (Table 1). There are differences in manpower requirements; for example, community-level health care centers do not require psychologists as a part of manpower. However, some community-level health care centers provide psychologist services.^11^

**TABLE 1:**
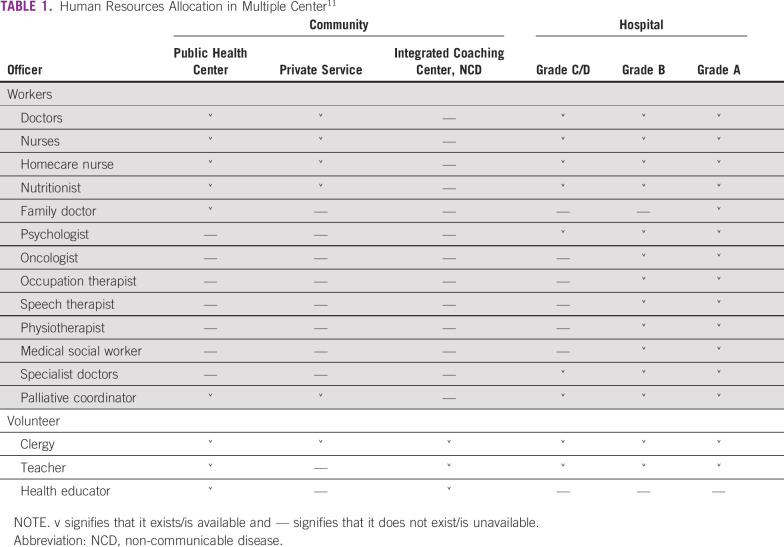
Human Resources Allocation in Multiple Center^11^

To monitor and evaluate program effectiveness for short- and long-term goals, the Ministry also developed a system to ensure that certain key performance indicator (KPI) were achieved. The evaluated items included patient demographics, prevalence, incidence, mortality, opioid usage, provision of homecare, socialization, education and training, and budgeting.^11^ Primary health care centers (*pusat Kesehatan masyarakat/puskesmas*) are considered the spearpoint through a family-centered approach.^25^

Jakarta and Surabaya provided an example of local government action in palliative care using a family approach. The district of Health DKI Jakarta as a capital city has a cancer burden with an estimated number of 16,929 patients in 2019, in which the majority are in an advanced stage in need of palliative care, including at home. In the palliative care program, 333 primary health care centers were trained to serve patients in need of the area, covering the entire capital city. In 2020, Jakarta *puskesmas* provided palliative care for 713 patients with cancer and 583 patients without cancer.^37^

Another example of integration is shown by Surabaya, the first city in Indonesia to develop palliative care in 1992 by publishing a mayor's decree to establish a city palliative team. Innovation in community development included capacity building and training for primary health care workers, family caregivers, and community volunteers (*kader*). Sixty-three *puskesmas* worked alongside professionals in tertiary care to deliver high-quality palliative care in the community. Care extended from hospital inpatient and outpatient units to home care.^38,39^

## DISCUSSION

Indonesia has unique modalities for overcoming the challenges of cancer palliative care implementation. ICF has unique positioning as nonprofit organization, but at the same time having strong ties and collaboration with government. ICF branches throughout Indonesia allow access to education in rural areas and care delivery through the training of the local community. These traits could serve as building blocks for the nationwide development of community- and home-based palliative care.

Another valuable resource is CHVs with initiatives in accordance with the local cultural and social value of mutual assistance (*gotong royong*). The Indonesian CHV, called *kader* effort, has continued for decades despite its noncommercial nature, thus having the potential for ongoing participation to improve community health care in the future. In palliative care implementation, trained CHV is a valuable asset for identifying individuals in need of care and continuing care when patients are sent back home to their family.

Homecare-based palliative care demand would increase in the future as a result of increasing cancer numbers; thus, training for larger numbers of CHVs should be the focus. However, considering that most CHVs may not be medically or formally trained in health care, the roles and types of care delivered may be limited. A balance is needed between the standardization of training and acknowledgment of certified CHVs in palliative care and the code of conduct or limitation of their authority.

Comparing the cancer burden in Indonesia and the number of patients receiving palliative care through the cancer foundation and primary health care centers, there are still many patients who have not received palliative care as needed. These issues may be related to the limited human resources, funding, and disparities between cities. Therefore, systematic continuous training, human resource recruitment, and funding commitment for future development are required to ensure the growth and scale of palliative care.

Considering that a national policy for palliative care integration in the community and tiered reporting systems has been established, future developments have the opportunity to grow. However, current data and reports of palliative care work in the community mainly come from the major cities of Jakarta, Surabaya, and other cities in the Java/Sumatra region. This indicates the need to create awareness, expand services, and provide outreach training for other islands and provinces in Indonesia. Effective scaling can be achieved through the collaboration and utilization of the current network of cancer foundations, national health care centers, and the national population system.

In conclusion, despite the challenges in implementing home-based palliative care in the Indonesian community, there are strengths and opportunities available for future development. Indonesia's model of public-government cooperation through the ICF, community initiative participation through CHVs, and government commitment to integrate community resources are valuable and effective models for Indonesia's large population.
